# Spectral sensitivity and temporal resolution of the sexually dimorphic compound eyes of *Electrogena lateralis*

**DOI:** 10.1038/s41598-025-14294-4

**Published:** 2025-08-12

**Authors:** Ádám Egri, Mihály Jásdi, György Kriska

**Affiliations:** 1 Institute of Aquatic Ecology, HUN-REN Centre for Ecological Research, Karolina út 29, Budapest, 1113 Hungary; 2 Fluvial Ecology Research Group, MTA-ÖK Lendület Momentum, Karolina út 29, Budapest, 1113 Hungary; 3 Group for Methodology in Biology Teaching, Biological Institute, Eötvös University, Pázmány Sétány 1, Budapest, 1117 Hungary

**Keywords:** Mayfly, Flicker fusion frequency, ERG, Visual ecology, Colour vision, Motion detection, Freshwater ecology

## Abstract

Several mayfly species have sexually dimorphic compound eyes. Female *Electrogena lateralis* have uniform eyes, while male eyes are split into ventral and dorsal regions. We compared the spectral sensitivity and temporal resolution of the male eye regions and female eyes using electroretinography. Larval spectral sensitivity was also measured. Temporal resolution was quantified by measuring the eye flicker fusion frequency. The male ventral eye region and female eye were most sensitive to the green spectral range, with major and minor peak sensitivities at 536 and 366 nm, respectively. The dorsal eye region of males was exclusively ultraviolet sensitive, with maximal sensitivity at 353 nm. Relative ultraviolet sensitivity of larvae was lower than that of adults, which could be explained with the transmittance of the exuvium. Flicker fusion frequency of the male dorsal eye region was significantly higher than that of the ventral region. Temporal resolution of female eyes was similar to that of the ventral eye region of males. The high temporal resolution of the dorsal eye region and the exclusive ultraviolet sensitivity in males might play a role in the detection of females during swarming, while the female eye and the ventral male eye region may serve general navigation.

## Introduction

The ability of vision is a fundamental capability of insects. The most typical eye design in insects is the compound eye^1^. Since the visual task of the different eye regions can be completely different, insect compound eyes are often regionalised^2^. Moreover, in certain species, the eye structure differs between females and males^3^. In mayflies, sexual dimorphism of the compound eyes is a known phenomenon^4^. Eyes of males are usually divided into a ventral and a dorsal region, which is most pronounced in the family Baetidae^5^. Males utilize their dorsal eye regions during swarming for detecting females appearing as dark spots in front of the bright background provided by the skylight^6^. Horridge and McLean^7^ showed that the dorsal eye of *Atalophlebia* mayflies is exclusively UV–sensitive which is in accordance with the short wavelength-rich spectrum of the twilight sky under which swarming takes place.

**Fig. 1:**
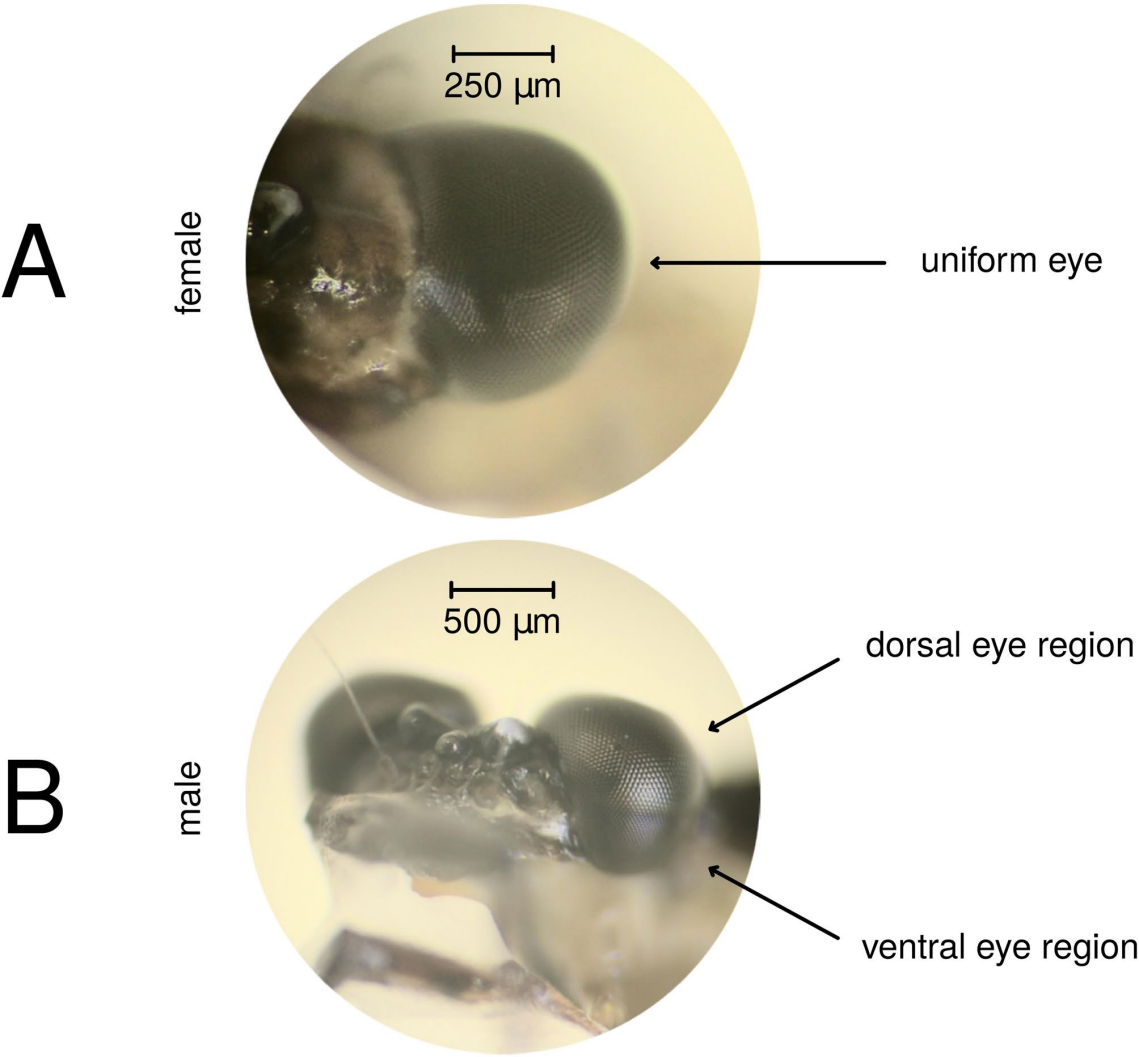
Photographs of adult *E. lateralis* compound eye preparations. (**A**) Frontal view of a female eye. (**B**) Fronto-lateral view of a male eye with the clearly visible dorsal and ventral eye regions.

In males of the heptagenid mayfly *Electrogena lateralis* (Curtis, 1834) [= *Heptagenia lateralis* (Curtis, 1834)] the compound eye is also split horizontally to a ventral and a dorsal eye region^8^. These regions have slightly different ommatidial sizes and colours, which are clearly visible when observed through a stereo microscope (Fig. 1A). In contrast, the female eyes are uniform and slightly smaller than the male eyes (Fig. 1B).

Compound eyes consist of multiple units called ommatidia, which contain photoreceptors sensitive to specific spectral ranges and often to polarized light^1^. Each ommatidium collects light from a given, relatively narrow field of view. The angle between the optical axes of the neighbouring ommatidia and the acceptance angle of the ommatidia greatly determine the spatial resolution of the compound eye^9^. The higher the spatial resolution of the eye, the more effectively the visual system can recognize small details. Besides spatial resolution, temporal resolution is also an important parameter of the eye that affects behavioural responses to visual stimuli^10^. In general, the temporal resolution of eyes of fast flying insects is higher than that of slower species, because the individual must be able to avoid obstacles and objects by their rapid visual detection^11^.

Electroretinography (ERG) is an electrophysiological method used, for example, to measure the spectral sensitivity of an insect eye^12^. Spectral sensitivity of mayfly eyes has been measured in only a few cases^7,13–15^, and we are not aware of any attempts to measure the temporal resolution of mayfly eyes. Inspired by Gupta et al.^16^, who studied the eyes of *Cloeon* mayflies, we hypothesized that the dorsal eye region of *E. lateralis* is mainly short wavelength-sensitive and the response of its photoreceptors is faster than that of the ventral eye region, because males must be able to grab females immediately when they pass through the field of view of the dorsal region of the male eye. The reason for choosing *E. lateralis* for our experiments was twofold. (i) This species can be easily collected and kept and (ii) the division of the male eye is not as strikingly obvious as it is in *Atalophlebia* species^7^, for example. Hence, we performed a series of electroretinography measurements on *E. lateralis* eye preparations. Our primary aim was to compare the spectral sensitivity and temporal resolution of the ventral and dorsal eye regions of the males, but females and larvae were also included as subjects in our study. Regarding larvae, our aim was to test whether their compound eye spectral sensitivity differs markedly from that of adults, which was recently demonstrated for the burrowing mayfly *Ephoron virgo* (Olivier, 1791) [= *Polymitarcis virgo* (Olivier, 1791)]^14^.

## Materials and methods

### Mayflies

Larvae of *E. lateralis* were collected from the Domini creek (Northern Hungary, N 47°40′6.898″ E 18°58′0.109″) and were kept in the laboratory in an aerated aquarium. Biofilm on field-collected stones provided food for the larvae, which emerged in 1–3 weeks after collection. The aquarium was placed in a cage, which prevented the emerged adults from escaping.

### ERG recordings

Mayflies were prepared on a piece of plexiglas. In the case of adults, the abdomen and partly the thorax with the wings were fastened to the plexiglas with adhesive tape, while the head and the forelegs were fixed to the plexiglas using melted paraffin wax. Abdomen of larvae was also attached to the plexiglas with adhesive tape, and a water droplet was injected around the gills beneath the tape. For fixing the larval mandibles, the same adhesive tape was used. Tungsten electrodes etched in saturated KNO_2_ solution were used for recording. The recording and reference electrodes were inserted to the right eye and the abdomen, respectively. Photoreceptor responses were amplified with a Model 3000 AC/DC Differential Amplifier (A-M Systems, Carlsborg, Washington, USA) and were recorded with a USB sound card (C-Media CM6206 chipset) modified for DC measurements. Recordings were made with Audacity 2.2.1 audio recording software (https://www.audacityteam.org) in 16-bit WAV format. The linearity of the sound card inputs was checked and verified within the ± 1 V range, therefore the output of the amplifier was kept far within this range. The left and right channels, respectively, were used for recording the amplified photoreceptor responses and a square wave reference signal indicating the presence of the light stimulus. The reference signal facilitated the detection of photoreceptor responses in the data and enabled us to calibrate the voltage level of the left channel.

### Spectral sensitivity

Spectral sensitivity of *E. lateralis* compound eyes was measured with the flash method^12^. Quasi-monochromatic light stimuli of different wavelengths and intensities were created with a custom-built LED-based light source^17^. The following wavelengths (± half bandwidth of LED) were used in the measurements: 346 nm (± 5.0 nm), 376 nm (± 4.8 nm), 402 nm (± 5.5 nm), 421 nm (± 6.4 nm), 442 nm (± 8.5 nm), 467 nm (± 10.4 nm), 496 nm (± 13.5 nm), 516 nm (± 14.5 nm), 552 nm (± 17.7 nm), 598 nm (± 6.9 nm), 623 nm (± 7.7 nm), 641 nm (± 8.6 nm). A Moritex SOHC4S3.5-1500S quartz light guide was used for delivering the light stimuli to the eye preparation.

Spectral sensitivity of adult female compound eyes and male ventral eye regions were performed on compound eyes adapted to darkness, to the light of a green (562 nm ± 23.8 nm; peak wavelength ± half bandwidth) and UV (377 nm ± 8.9 nm) LED. The dorsal eye region of males was measured only dark-adapted. Spectral sensitivity of female and male larvae was measured on dark- and green-adapted individuals. Dark-adaptation was at least 40 min long. Although in the case of larvae no eye regions could be distinguished visually, recording electrodes were inserted to the lower eye region. Photon flux of the adapting light was 1.4 × 10^14^ photons cm^−2^ s^−1^ and the optical axis of the light guide encompassed approximately 20° with the light stimulation. Chromatic adaptation was 15 min long and was often applied on previously dark-adapted eye preparations, the spectral sensitivity of which was just measured. Numbers of measurement types are summarised in Table 1.

**Table 1:** Number of spectral sensitivity measurements performed on dark- and chromatic-adapted *E. lateralis* eye preparations of different life stages and sexes.

Life stage	Sex	Eye region	Adaptation type	N
Adult	Female	–	Dark	10
Adult	Male	Ventral	Dark	9
Adult	Male	Dorsal	Dark	6
Adult	Female	–	Green	5
Adult	Female	–	UV	5
Adult	Male	Ventral	Green	5
Adult	Male	Ventral	UV	5
Larva	Female	–	Dark	6
Larva	Male	–	Dark	3
Larva	Female	–	Green	3
Larva	Male	–	Green	1

In the case of males, both ventral and dorsal eye regions were separately examined.

Measurement of a given eye preparation consisted of the repetition of the same stimulus sequence during which the applied wavelengths were in increasing then in decreasing order. In the case of a given wavelength the photon flux of the stimuli was logarithmically increasing (step ≈ 0.5 log unit). The light stimuli were 500 ms long separated with 6-s-long dark inter-stimulus periods. When wavelength was switched, 12-s-long interstimulus period was applied. The stimulus sequence was repeated typically 3 times. Photon flux of the applied stimuli ranged between 2.0 × 10^12^ and 2.1 × 10^16^ photons cm^−2^ s^−1^. Recordings were checked and those were excluded from further analysis in which the overall appearance of the repetitions differed.

**Fig. 2:**
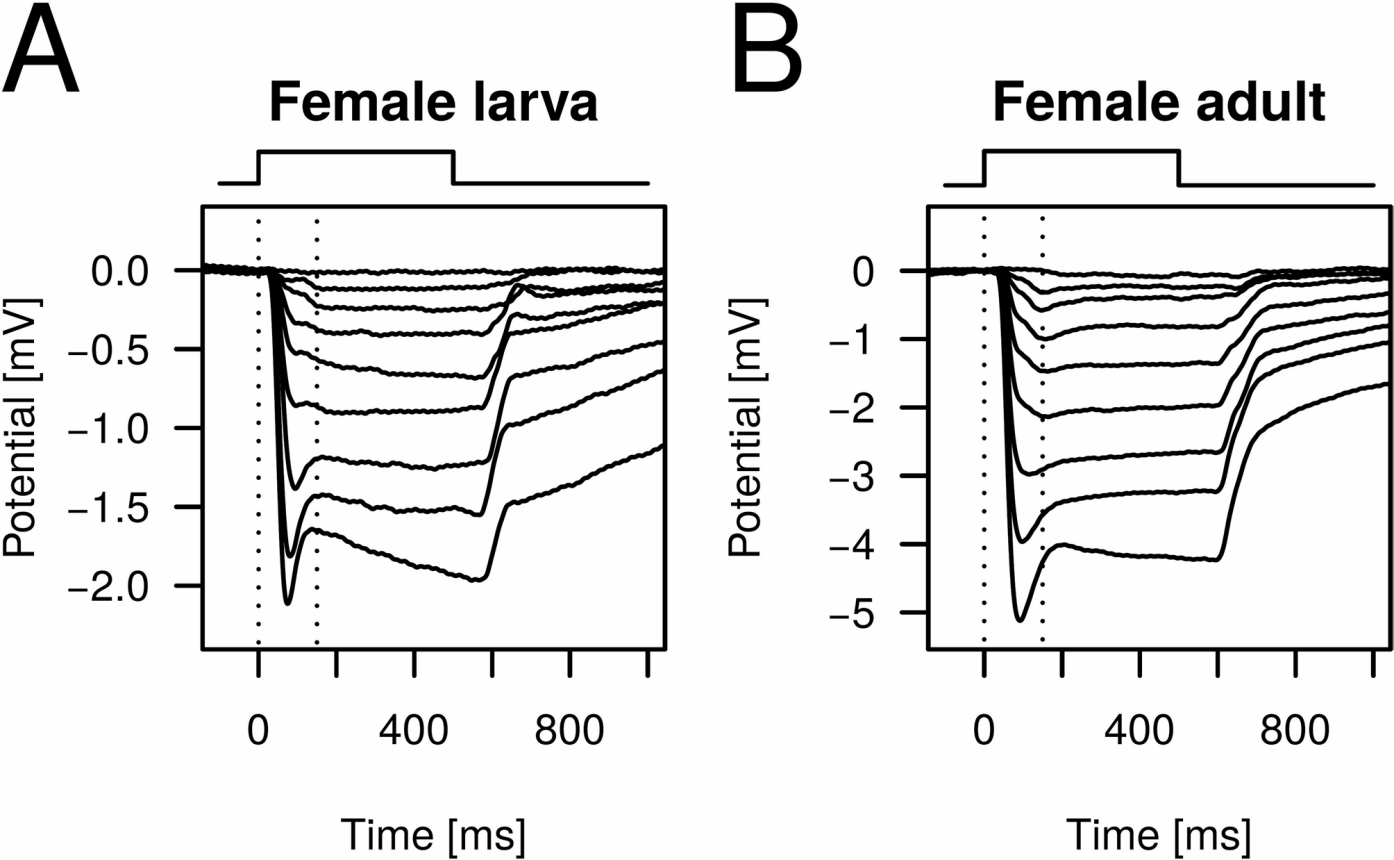
Example photoreceptor responses elicited by 442 nm light stimuli of logarithmically increasing photon flux (2.1 × 10^12^ photons cm^−2^ s^−1^ < I < 2.1 × 10^16^ photons cm^−2^ s^−1^; step ≈ 0.5 log unit). Rectangular pulses above each graph show the presence of light stimulus. Pairs of vertical dotted lines show the first 150 ms of the stimulus in which the response amplitude was determined. (**A**) Responses of a female larva. (**B**) Responses of a female adult.

For each light stimulus the amplitude of the photoreceptor response was considered as the magnitude of the negative jump in potential in the first 150 ms of the stimulus (Fig. 2). For each wavelength the response amplitudes were plotted against log stimulus photon flux, Naka-Ruhston function^18,19^ was fitted on the points and the stimulus intensity needed for eliciting a critical response amplitude (20% of the maximal response amplitude measured for the given preparation) was calculated. The reciprocals of these critical stimulus intensities resulted in the spectral sensitivity curve, which was finally normalized with the value at 516 nm. In the case of male dorsal eye regions, the value at 346 nm was used for normalization. Relative spectral sensitivities were modeled by fitting the sum of two Govardovskii templates^20^, except for the male dorsal preparations for which a single template was fitted. All curve fittings were performed using the Levenberg–Marquardt Nonlinear Least-Squares Algorithm (minpack.lm R package)^21^.

Relative spectral sensitivity of dark-adapted female and male ventral eye preparations were compared wavelength-by-wavelength with Wilcoxon Rank Sum tests. As Govardovskii templates were fitted to the normalized spectral sensitivity of every single preparation, fitted sensitivity peaks of the assumed UV and green receptors were also compared with Wilcoxon Rank Sum tests for female and male ventral eyes. In the case of female eyes and male ventral eye regions, relative spectral sensitivity of the UV- and green-adapted preparations were compared with Wilcoxon Rank Sum tests. Spectral sensitivity of dark-adapted female and male larval eyes, and female dark- and green-adapted eyes were also compared wavelength-by-wavelength with Wilcoxon Rank Sum tests, as well as the relative spectral sensitivity of dark-adapted adults and larvae with pooled female and male data.

### Flicker fusion frequency

Flicker fusion frequency (FFF) is the critical frequency for which the photoreceptor responses elicited by distinct flashes of light become merged into a single continuous response^22^. FFF of *E. lateralis* female eyes and both eye regions of males was measured by determining the highest frequency at which unambiguous modulation of the photoreceptor response could still be elicited by stroboscopic light stimuli. Arduino-controlled LEDs were used for creating flickering stimuli. Because spectral sensitivity measurements performed on chromatic-adapted preparations revealed no more than two distinct photoreceptor classes, FFF measurements were made at two wavelengths, 520 nm (green) and 372 nm (UV). Since male dorsal eyes were only UV–sensitive, only UV stimulation was applied to these preparations. For a given wavelength, stimuli of 25 frequencies were applied. These frequencies were logarithmically spaced between 5 and 150 Hz. Stimuli were 2 s long separated by 10-s-long dark inter-stimulus periods. First, green, then UV stimuli were applied, then the whole sequence was repeated 3 times, thus a total number of 3 stimuli were performed for a given wavelength and frequency. Mean light intensity of the UV and green flickering stimuli were 1.2 × 10^15^ and 2.0 × 10^15^ photons cm^−2^ s^−1^, respectively. During FFF recordings the filters of the amplifier were set to pass frequencies between 1 Hz and 3 kHz and the 50 Hz notch filter was switched off.

**Fig. 3:**
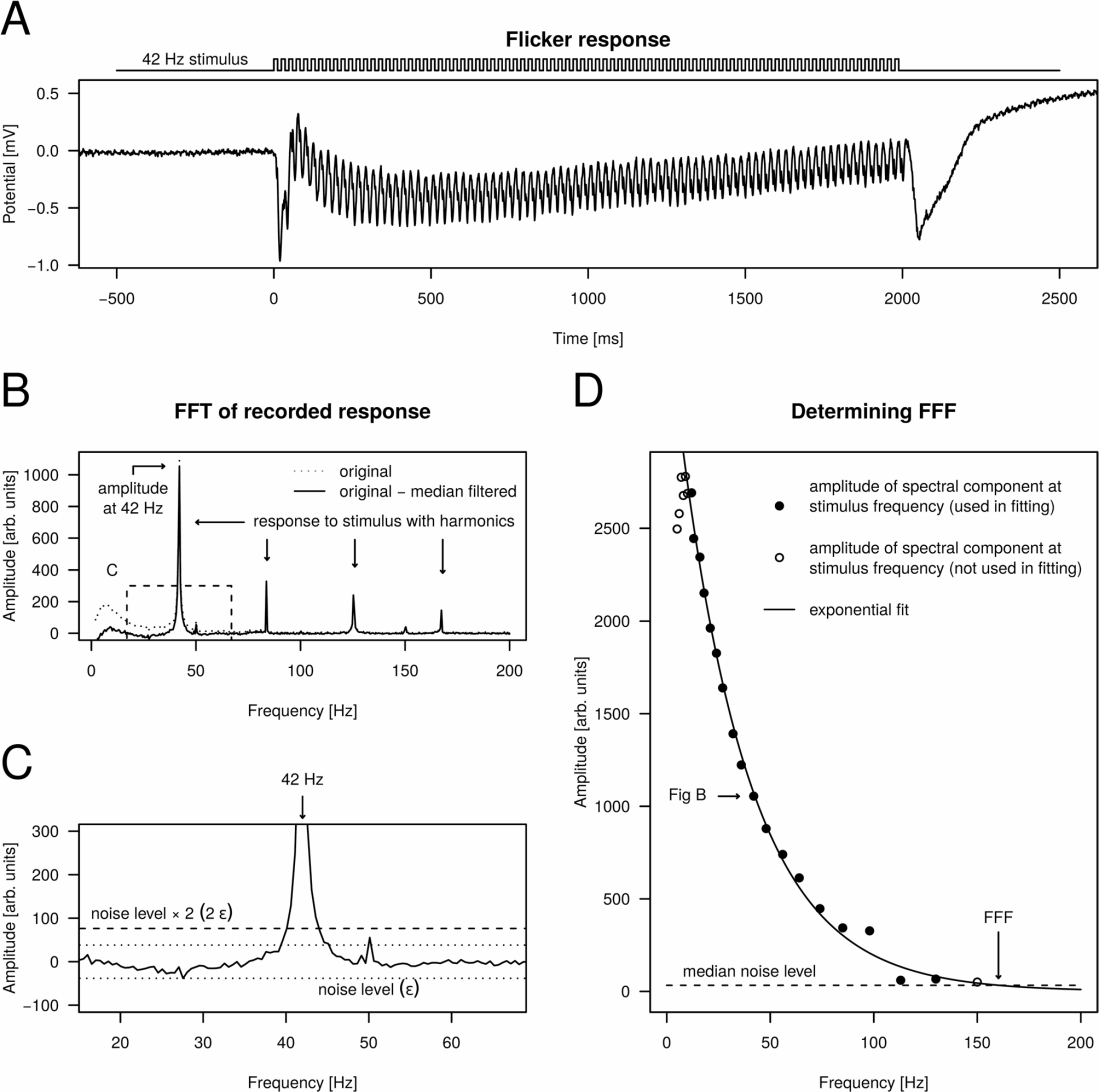
Calculation of the flicker fusion frequency. (**A**) Example photoreceptor response to a 42 Hz stroboscopic stimulus (λ = 372 nm) recorded from the dorsal eye region of a male *E. lateralis.* (**B**) Amplitude spectrum of the recorded response obtained by calculating the fast Fourier transform of the signal shown in A. Dotted curve: original amplitude spectrum; solid curve: median-filtered variant (window width = 50 Hz) subtracted from the original spectrum. The dashed rectangle shows the ± 25 Hz neighbourhood of the stimulating 42 Hz. (**C**) Enlarged view of the ± 25 Hz neighbourhood of the stimulating 42 Hz in the amplitude spectrum (dashed rectangle in B) for deciding whether the stimulating frequency elicited a clear response. (**D**) Amplitude spectrum values at stimulating frequencies plotted for all applied stimulus frequencies with the fitted decaying exponential. Solid points: stimulation elicited clear response (amplitude > 2ϵ); empty points: stimulation did not elicit a clear response (amplitude ≤ 2ϵ) or manual exclusion (for very low stimulating frequencies). The horizontal dashed line represents the median noise level obtained by calculating the median of noise levels for all stimulating frequencies (graphs like **C**) that elicited a response (solid points in **D**). The intersection of the exponential and the median noise level provided the FFF estimating the frequency for which the response was lost in the noise.

To determine the FFF for a given preparation, we performed the following steps. Before each recording, the preparation was dark-adapted for 40 min. In the case of a given wavelength, for each stimulating frequency ${f}_{stim}$ the recorded photoreceptor response during the 2-s-long stimulus (Fig. 3A) was Fourier transformed, and the amplitude spectrum was further analyzed (Fig. 3B). The amplitude spectrum was adjusted by subtracting its median-filtered variant (window width = 50 Hz), which resulted in a flat baseline around zero (Fig. 3B). Next, the presence of the stimulating frequency in the recorded signal was tested by searching for a peak in the amplitude spectrum at the stimulating frequency (Fig. 3C). This was achieved by defining the noise level in the amplitude spectrum around the stimulating frequency within a 25 Hz radius. The noise level $\epsilon$ was the absolute value of the most negative value within the ± 25 Hz neighbourhood of ${f}_{stim}$ (Fig. 3C). If the value of the amplitude spectrum at ${f}_{stim}$ was greater than $2\epsilon$, a clear response was confirmed, thus the stimulation was considered as being sensed by the eye. Finally, for all responses, amplitude spectrum values at the corresponding stimulating frequency were plotted against ${f}_{stim}$ and exponential decay function was fitted to the points for which a clear response was confirmed (Fig. 3D). In other words, the modulation amplitude in the responses caused by the stroboscopic stimuli were plotted as a function of stimulating frequency. At last, FFF was obtained by taking the frequency where the fitted exponential curve intersected the median noise level (Fig. 3D). The median noise level was obtained by calculating the median of $\epsilon$ values originating from the stimuli for which a clear photoreceptor response was confirmed.

Since three repetitions of the stimulus sequence were performed for both tested wavelengths, three FFF values were acquired per wavelength. The final FFF value corresponding to a specific wavelength was calculated by averaging these 3 values. A total number of 6 female, 9 male ventral and 9 male dorsal eye preparations were tested.

FFF of female compound eyes obtained for the green and UV stimulation were compared with Wilcoxon Rank Sum test. We used pairwise Wilcoxon Rank Sum tests with Bonferroni’s correction to reveal FFF differences between the green- and UV–stimulated ventral eye and the UV–stimulated dorsal eye.

## Results

### Spectral sensitivity

**Fig. 4:**
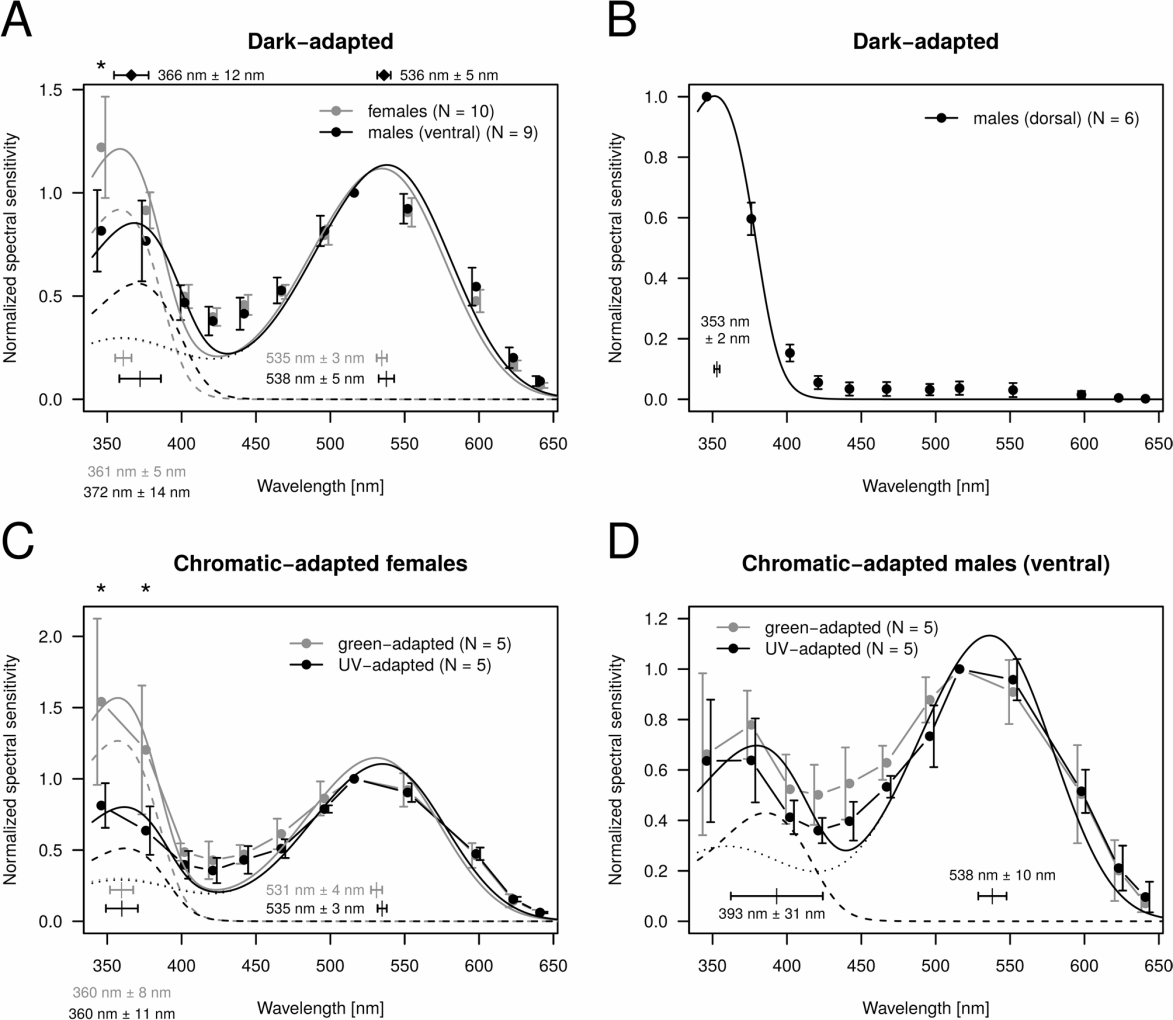
Mean normalized spectral sensitivity of adult compound eyes with vertical bars denoting SD. (**A**) Dark-adapted female (gray) and male ventral eye preparations (black). Continuous curves show the fitted sum of two Govardowskii templates (dashed curve: template for the supposed UV receptor; dotted curve: template for the green receptor). Separately for females and males, and for the supposed UV and green sensitive photoreceptors, horizontal error bars within the graph show the mean ± SD of sensitivity peak wavelengths obtained for all measured preparations. Mean ± SD of sensitivity peak wavelengths after pooling female and male data together are shown by horizontal error bars above the graph. For each indication of the mean and SD, the corresponding numerical values are also shown. The asterisk shows a significant (α = 0.01) sensitivity difference between females and males revealed by the Wilcoxon Rank Sum test at 346 nm. (**B**) Dark-adapted male dorsal eye preparations with a fitted template. The narrow wavelength range shown by the horizontal error bars below the curve indicates the mean ± SD of the sensitivity peak wavelengths of the individual preparations. (**C**) Green- (gray) and UV–adapted (black) female eye preparations. Asterisks show significant (α = 0.01) sensitivity differences revealed by Wilcoxon Rank Sum test. (**D**) As C for male ventral eye preparations. Templates were fitted on the pooled data of females and males.

Dark-adapted male ventral and female eyes exhibited primary sensitivity to the green, with a secondary sensitivity peak in the UV spectral range (Fig. 4A). Sensitivity of females and males were statistically similar at all wavelengths, except for 346 nm at α = 0.01 significance level. The sum of two visual pigment templates was fitted to the spectral sensitivity of each preparation, providing an estimation for the sensitivity peak of hypothetical green and UV photoreceptors. According to Wilcoxon Rank Sum tests, the locations of sensitivity peaks of the assumed green and UV photoreceptors were statistically similar (UV: W = 68, *p* = 0.065; green: *W* = 64, *p* = 0.133). Therefore, after pooling together the male ventral and female sensitivity peaks, the sensitivity maxima of the UV and green receptors were finally determined at wavelengths of 366 nm ± 12 nm and 536 nm ± 5 nm (mean ± SD), respectively. The dorsal eye region of males was exclusively UV–sensitive, with an average sensitivity peak at 353 nm ± 2 nm (mean ± SD) (Fig. 4B).

Recordings on chromatic-adapted female eye preparations confirmed the presence of distinct UV–and green-sensitive receptors. According to wavelength-by-wavelength Wilcoxon Rank Sum tests, spectral sensitivity of green-adapted preparations possessed significantly (α = 0.01) higher relative sensitivity in the UV range than the UV–adapted female individuals (Fig. 4C). When comparing chromatic-adapted male ventral eye preparations, no statistically significant differences in relative sensitivity were observed at any wavelength (Fig. 4D).

**Fig. 5:**
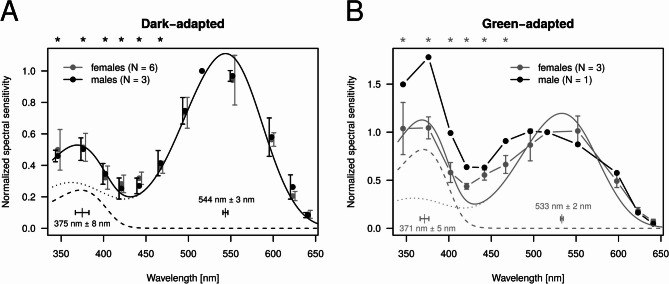
Mean normalized spectral sensitivity of larval compound eyes with vertical bars denoting SD. (**A**) Dark-adapted female (gray) and male eye preparations (black). The continuous curve shows the sum of two Govardowskii templates fitted on the pooled spectral sensitivity data of females and males (dashed curve: template for the supposed UV receptor; dotted curve: template for the green receptor). Separately for the supposed UV and green-sensitive photoreceptors, horizontal error bars within the graph (with numerical values) show the mean ± SD of sensitivity peak wavelengths obtained for all measured preparations. Asterisks show significant (α = 0.01) sensitivity differences revealed by Wilcoxon Rank Sum tests between the dark-adapted adult (Fig. 4A) and dark-adapted larval (Fig. 5A) eye preparations. (**B**) Green-adapted female (gray) eye preparations and a single male eye preparation (black). Asterisks show significant (α = 0.05) sensitivity differences revealed by Wilcoxon Rank Sum tests between the dark-adapted (gray curve in **A**) and green-adapted (gray curve in **B**) female larval eye preparations.

In the case of dark-adapted larval eyes, Wilcoxon Rank Sum tests detected no significant (α = 0.05) difference between females and males at any wavelength (Fig. 5A). Thus, data of females and males were pooled together. After fitting the sum of two Govardowskii templates on the data of each eye preparation, we obtained 375 nm ± 8 nm and 544 nm ± 3 nm (mean ± SD) for the sensitivity peak wavelength of the hypothetical UV and green receptors, respectively. Green adaptation on female larvae resulted in increased relative spectral sensitivity in the 346–467 nm range (Fig. 5A, B), which is the clear indication of distinct UV–and green-sensitive photoreceptors already in the larval stage. The single examined green-adapted male larval eye preparation was undoubtedly more sensitive to the UV range than the green adapted females (Fig. 5B). The relative spectral sensitivity of adults was significantly (α = 0.01) higher than that of larvae in the 346–467 nm range (Fig. 5A).

### Flicker fusion frequency

**Fig. 6:**
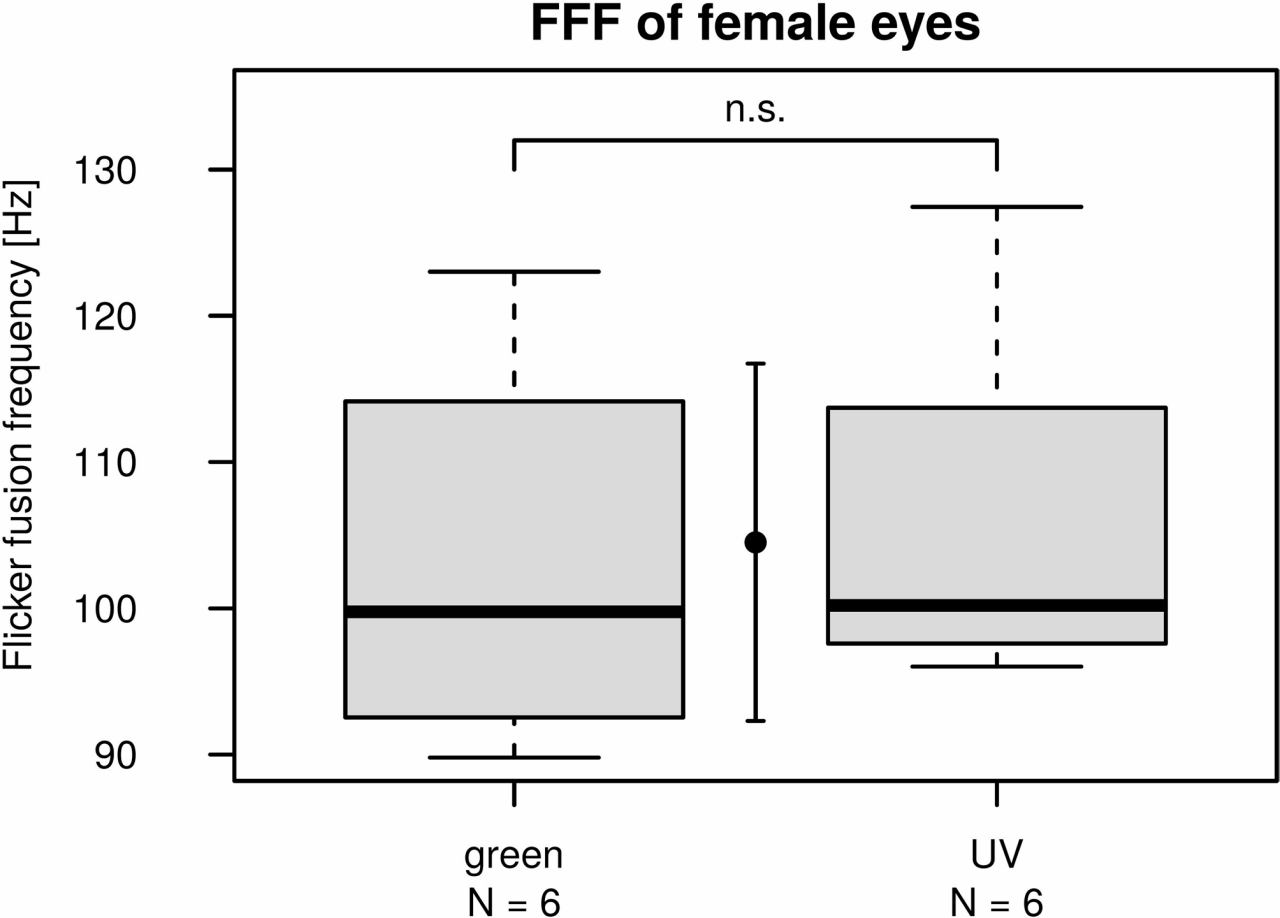
Boxplots of flicker fusion frequencies of female *E. lateralis* eyes measured with green and UV stroboscopic stimuli. The point with the vertical bars between the boxes shows the mean ± SD of all obtained FFF values pooled together.

The FFF measured in female eyes with UV and green stimulation did not differ significantly according to the Wilcoxon Rank Sum test (W = 22, *p*-value = 0.5887). Thus, we can consider 104.5 Hz ± 12.2 Hz as the flicker fusion frequency of dark-adapted female eyes being the mean ± SD of all calculated FFF values (Fig. 6).

**Fig. 7:**
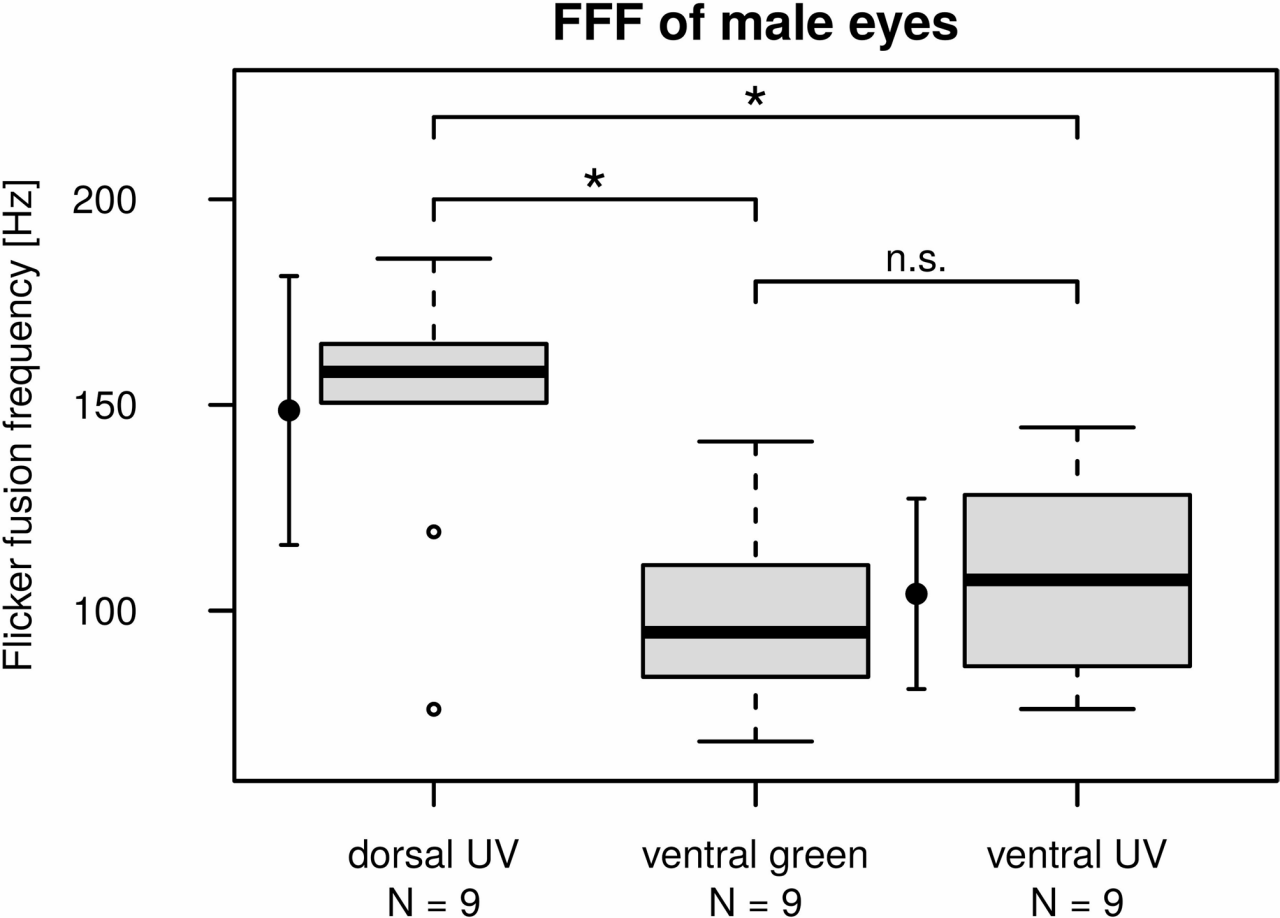
Boxplots of flicker fusion frequencies of dorsal and ventral eye regions of male *E. lateralis*. Ventral eye regions were examined with green and UV stroboscopic stimuli, while dorsal regions were only stimulated with UV light. Asterisks above the boxes indicate significant differences revealed by pairwise Wilcoxon Rank Sum tests at α = 0.05 significance level. The point with the vertical bars on the left shows the mean ± SD of FFF obtained for the dorsal region. The mean ± SD calculated for the pooled FFF values of green- and UV–stimulated ventral eye regions are shown by the point and vertical bars between the corresponding boxes.

According to the pairwise Wilcoxon Rank Sum tests, FFF values of the male dorsal eyes were significantly higher than that of the ventral eyes irrespective of the stimulation wavelength (dorsal vs ventral (green): *p* = 0.017; dorsal vs ventral (UV): *p* = 0.032). Mean ± SD of the FFF values obtained for the dorsal eyes was 148.7 Hz ± 32.7 Hz. Because no significant difference was detected between the FFF of the ventral eyes obtained for green and UV stimulation (*p* = 1), we consider the mean ± SD of FFF values of the green- and UV–stimulated ventral eyes, being 104.1 Hz ± 23.2 Hz, as the FFF of the ventral eye region (Fig. 7).

## Discussion

In nature the only relevant source of ultraviolet illumination is the Sun, the light of which being scattered in the atmosphere forms the skylight^1^. The exclusive UV sensitivity of the dorsal eye region in *E. lateralis* males is most likely an adaptation to the optical environment during mating because males of heptagenid mayflies seek females in front of the bright background provided by the sky^6^. Exclusive UV sensitivity was already reported for the dorsal eye of *Atalophlebia* mayflies, the swarming behaviour of which is similar to that of *E. lateralis*^7,13^. On the other hand, the spectral sensitivity of the ventral eye region in *E. lateralis* is characterized by a major sensitivity peak in the green range, which is again similar to the spectral sensitivity of the lateral eye in *Atalophlebia* mayflies^13^. Because the majority of photoreceptors in a variety of insect eyes are green-sensitive^23^, we suggest that the ventral eye region of *E. lateralis* plays a role in general navigation tasks, where the background is often dominated by longer wavelengths (e.g. green vegetation and soil) compared to skylight.

Regarding their egg-laying behaviour, heptagenid mayflies, like *E. lateralis* are “dippers”, which means that females fly upstream just above the creek and frequently dip their abdomen into the water, each time releasing a few eggs^24^. Therefore, for females, it is essential to effectively locate the water surface. Similar to other aquatic insects, certain mayflies optically locate water surfaces by means of the water-reflected horizontally polarized light^25–27^. Because polarisation vision in aquatic insects tend to operate in the UV or blue spectral region^28^, it is reasonable to assume that the distinct UV–sensitive photoreceptors in the compound eyes of *E. lateralis* females, revealed by our recordings on chromatic-adapted individuals, could account for polarisation vision. We could also confirm the presence of UV–sensitive photoreceptors also in female larvae.

Involving larvae in our experiments resulted in the second ever measurement of the larval compound eye spectral sensitivity of a mayfly species. In a former study we showed that the relative UV sensitivity of *E. virgo* adults is remarkably higher compared to that of the larvae^14^. This difference could not be explained solely with the transmission spectrum of the larval skin, which is left behind by the emerging adult. In the present study we found that the relative UV sensitivity of *E. lateralis* adults (Fig. 4A) was also significantly higher than that of larvae (Fig. 5A). However, unlike in *E. virgo*, this difference can be explained by the transmittance properties of the larval skin, which covers the eye during the larval stage. Corneal transmittance measurements on larval skins of the mayfly *E. virgo* and the dragonfly *Libellula depressa* Linnaeus, 1758 indicated that compared to green light, approximately 20% less UV light is transmitted through the exuvium towards the eye^14^. Consequently, after casting off the larval skin during emergence, the amount of UV light entering the eye increases, resulting in a higher measured relative sensitivity. Assuming similar transmittance characteristics for the larval skin of *E. lateralis*, we propose that the spectral sensitivity of the adult compound eye is qualitatively the same as that of the larvae beneath the larval skin.

Pronounced or exclusive dorsal UV sensitivity is widespread among insects, but its function is not limited to mate recognition, as it is in several mayfly species^5^. For example, the dorsal rim areas of bee and cricket eyes, specialized for skylight polarisation-based navigation are also exclusively UV–sensitive^1^. To mention other examples, in *Sympetrum* dragonflies and in black flies (Simuliidae), prey detection in front of the background provided by the sky is facilitated by the dorsal eye region, which is blue- and UV–sensitive in the former^29^ and exclusively UV–sensitive in the latter^2^.

Visual detection of prey or mates in flight requires a sufficiently fast visual system, which can be characterized by the flicker fusion frequency of the eye. Since different eye regions often serve distinct visual tasks, compound eyes are usually regionalised in terms of temporal resolution as well. For example, in male hoverflies, the response time of photoreceptors in the so-called frontal “love spot” is 60% faster than in the surrounding eye regions, the purpose of which is the detection of females^30^. In the present study we found that the dorsal eye region of *E. lateralis* is analogous to the “love spot” of hoverflies. We hypothesize that the temporal resolution of the dorsal eyes and eye regions in other mayflies, including *Atalophlebia* species^7^ is also higher than that of the ventral/lateral eye regions.

Our FFF measurements were performed on dark-adapted eye preparations. It is well-known that FFF depends on the light adaptation state of the eye. FFF of a light-adapted eye can be 40–70% higher than that of the same eye in dark-adapted state^31,32^ and FFF increases with the intensity of the stimulating light^22,33,34^. Thus, in a real-life situation during swarming, compared to our results, remarkably higher temporal resolution may be present in both females and males of *E. lateralis*. For the exclusively UV–sensitive dorsal eye of *Atalophlebia* males it was shown that yellow pigments around the crystalline cone ensure an apposition type structure for the compound eye^7^. However, these pigments are more or less transparent to long wavelength light, which facilitates the transformation of metarhodopsin back to rhodopsin and allows for higher FFF^35^. When observed through a stereo microscope, the dorsal eye region of *E. lateralis* is also strikingly brownish compared to the dark ventral eye region. Thus, regarding the FFF ratio of the dorsal and ventral eye regions in males, we expect that the higher FFF in the dorsal region still persists when the eye is exposed to skylight during swarming.

### Summary

Similar to many other mayfly species, males of *E. lateralis* possess regionalised compound eyes. The male eye is divided into a ventral and a dorsal region, between which we identified functional differences. The dorsal eye region is exclusively UV–sensitive, and its temporal resolution is significantly higher than that of the ventral eye region. The spectral sensitivity and temporal resolution of female eyes and the male ventral eye region were qualitatively similar, both being most sensitive to green light. The relatively higher temporal resolution of the male dorsal eye region suggests a role in mate detection during swarming, while the female eyes and male ventral eye regions may serve general navigation. Larval UV sensitivity was lower than that of adults, likely due to the exuvium’s transmittance properties.

## Data Availability

The datasets generated during and/or analysed during the current study are available from the corresponding author on reasonable request.
